# Electromyographic Activity of Cervical Muscles in Patients with Neck Pain and Changes After Dry Needling: A Narrative Review

**DOI:** 10.3390/jcm13237288

**Published:** 2024-11-30

**Authors:** Julián Müller-Thyssen-Uriarte, María Orosia Lucha-López, César Hidalgo-García, Rocío Sánchez-Rodríguez, Lucía Vicente-Pina, Loreto Ferrández-Laliena, Pierre Vauchelles-Barré, José Miguel Tricás-Moreno

**Affiliations:** Unidad de Investigación en Fisioterapia, Spin Off Centro Clínico OMT-E Fisioterapia SLP, Universidad de Zaragoza, Domingo Miral s/n, 50009 Zaragoza, Spain; 732751@unizar.es (J.M.-T.-U.); 739468@unizar.es (R.S.-R.); l.vicente@unizar.es (L.V.-P.); lferrandez@unizar.es (L.F.-L.); pierrevauchelles@gmail.com (P.V.-B.); jmtricas@unizar.es (J.M.T.-M.)

**Keywords:** neck pain, myofascial trigger point, electromyography, dry needling

## Abstract

Neck pain can be associated with specific conditions, such as neurological disorders, vascular or inflammatory diseases, fractures, herniated discs, etc. However, the majority of neck pain cases cannot be attributed to a specific cause. The objective of this review is to describe the muscle dysfunctions associated with neck pain, as measured by electromyography, and to determine the effectiveness of dry needling in improving these muscular dysfunctions. The research was conducted using the following databases: PubMed, Alcorze, and Google Scholar. The next conclusions have been extracted after the revision of the 65 selected manuscripts. The current scientific evidence supports electromyographic pathological findings in individuals with chronic neck pain, especially during general upper limb movement, repetitive work, violin playing, cervical force, and cervical movement tasks. Dry needling applied to an active myofascial trigger point in the upper trapezius can be suggested as an intervention to enhance the performance in the cranio-cervical flexion. Dry needling applied to latent myofascial trigger points in the upper trapezius after typing tasks in healthy subjects resulted in decreased upper trapezius activity and fatigue in the short term. In women with trapezius myalgia, dry needling applied to the upper trapezius led to a lower increase in electromyography activity compared to no intervention.

## 1. Introduction

### 1.1. Neck Pain Definitions

Neck pain (NP) can be associated with specific conditions, such as neurological disorders, vascular or inflammatory diseases, fractures, herniated discs, etc. However, the majority of NP cases cannot be attributed to a specific cause, leading to the classification of non-specific neck pain (NSNP) [1]. NSNP, also referred to as mechanical NP, is defined as cervical pain (with or without radiation) without an identifiable pathological basis as the underlying cause of the complaint [2].

### 1.2. Prevalence of Neck Pain

The 1-year prevalence of NP has been estimated to range from 16.7–75.1% in the general adult population between 17 and 70 years of age, with a mean of 37.2% [3]. In Spain, there is a high prevalence of NP. According to data obtained from the Spanish National Health Survey, the 1-year prevalence of NP was 19.5%, with a higher frequency observed in females [4].

### 1.3. Costs and Socio-Economic Consequences of Neck Pain

Neck pain incurs treatment costs, reduced productivity, and job-related concerns. In 2016, among the 154 conditions studied by Dieleman and colleagues in the USA, NP combined with low back pain accounted for the highest healthcare expenses, estimated at USD 134.5 billion. Additionally, from 1999 to 2008, there was an increase in annual spending on care of approximately USD 487 to USD 950 per patient. For NP, 57.2% of costs were covered by private insurance companies, 33.7% by public insurance, and 9.2% through out-of-pocket payments in the USA. Other estimates, such as those from the Netherlands, indicated that NP represented a total cost of USD 686 million per year. Among all the health conditions studied, NP disorders accounted for one of the highest expenditures by private insurance [5].

The results of the study by Hoy et al. [6] indicate that the prevalence and burden of neck pain are high worldwide. Among the 291 conditions examined in the Global Burden of Disease 2010 study, NP ranked 21st in terms of overall burden and 4th in terms of overall disability.

### 1.4. Trigger Point Definition

The Delphi study for the international consensus on diagnostic criteria and clinical considerations for myofascial trigger points (MTrPs) [7] recommends that a minimum of two of the following three criteria should be met for the diagnosis of MTrPs: the presence of a taut band, a hypersensitive nodule, and referred pain. From a clinical perspective, we can differentiate between active and latent MTrPs. Local and referred pain originating from active MTrPs reproduces the symptoms reported by patients, and these patients recognize this pain as their usual discomfort, with which they are familiar [8].

### 1.5. Prevalence of Myofascial Trigger Point in Neck Pain

There is evidence that active MTrPs are more prevalent in individuals with mechanical NP compared to healthy subjects (HS) [9].

### 1.6. MTrPs and Electromyographic Activity

Abnormal endplate activity, commonly reported as spontaneous electrical activity near MTrPs in muscles, has been detected using electromyography (EMG) [10]. Electrical activity at the MTrPs has been correlated with pain intensity and pressure pain sensitivity [11]. One potential mechanism explaining the correlation between pain intensity and the increased amplitude of electrical activity may be related to heightened sympathetic activity at both latent and active MTrPs [12], which in turn increases motor unit activity.

It has also been found that intramuscular EMG amplitude of latent MTrPs was higher in the trapezius muscle compared to non-MTrP locations during an isometric shoulder abduction [13]. However, differences were not found in the superficial activity of the muscle at those locations. Higher amplitude of spontaneous electrical activity (SEA) at latent MTrPs when the muscle is at rest has also been noticed [14].

### 1.7. Dry Needling Definition

The term “dry needling” (DN) refers to the insertion of thin monofilament needles without the use of any substances [15], in contrast to the more commonly known “wet needling” techniques, which utilize hollow-bore needles to administer anesthetics, anti-inflammatories, or other substances [16]. Typically, target tissues for DN include muscles, fascia, neurovascular bundles, ligaments, scar tissue, and tendons in the treatment of various neuromusculoskeletal disorders.

The National Physical Therapy Association [16] in the United States defines DN as an “intramuscular” procedure targeting taut bands of muscle, commonly referred to as trigger points or MTrPs. This definition also encompasses the stimulation of neural, muscular, and connective tissues, not limited to just MTrPs.

DN is indicated for the treatment of patients with MTrPs that may lead to dysfunctions in body structure, pain, and disability. DN is also indicated when there are restrictions in range of motion caused by muscle fibers, taut bands, fascial adhesions, or scar tissue [16].

### 1.8. Dry Needling Mechanical, Physiological, and Neuromuscular Effects

DN modifies the chemical environment of active MTrPs, reduces or eliminates endplate noise, and decreases MTrP sensitivity [17]. DN can improve some parameters of neuromuscular function, such as tone, relaxation, creep, and pressure pain [18].

Some studies have reported the significant physiological effects of dry needling in patients with myofascial pain, such as increased muscle blood flow and oxygenation, as well as a decrease in nociceptive substances [19].

From a mechanical perspective, it has been suggested that trigger point dry needling can disrupt dysfunctional endplates, increase sarcomere length, and reduce the overlap between actin and myosin filaments [20]. Moreover, some studies have confirmed it can reduce the amplitude and frequency of endplate noise and endplate spikes, which are typical features of spontaneous electrical activity in trigger points, and it also reduces the response of the neuromuscular junction by decreasing the levels of the neurotransmitters [21].

From a neurophysiological perspective, dry needling (DN) mediates effects at various levels of the nervous system. Current evidence suggests that trigger point dry needling involves peripheral, spinal, and supraspinal mechanisms that underlie its effects. Specifically, the application of dry needling can reduce excitability by decreasing peripheral nociception at the trigger point, lowering dorsal horn neuron activity, and modulating brainstem regions. However, these effects are generally short-term, with small effect sizes, emphasizing the specific role that trigger points play in the complex experience of chronic pain [22].

### 1.9. Dry Needling General Clinical Effectiveness

Several pathologies can be effectively treated with DN. The effectiveness of this procedure has been measured by the reduction in pain and disability in conditions such as knee and hip osteoarthritis [23,24], neck pain [25], tension-type headaches [26], low back pain, shoulder pain, piriformis syndrome, carpal tunnel syndrome, plantar fasciitis, and temporomandibular disorders [27]. This procedure has proven effective in targeting MTrPs as well as neural and connective tissues, either in combination or when applied to neural and connective tissues alone [27].

### 1.10. Clinical Guidelines for Neck Pain and Current Evidence on Physical Therapy Treatments

On the one hand, some clinical guidelines provide relevant evidence regarding therapeutic recommendations for physical therapy in the management of neck pain. For example, a recent clinical guideline [28] based on scientific literature published prior to August 2016 recommends, with moderate strength of evidence, a multimodal approach for patients with chronic neck pain and mobility deficits. This approach should include cervical and thoracic manipulation or mobilization, exercises focusing on coordination, proprioception, and postural training, as well as stretching, strengthening, aerobic conditioning, and cognitive-affective elements. Additionally, treatments such as dry needling, laser therapy, and intermittent mechanical or manual traction are recommended. For patients with chronic neck pain and movement coordination impairments, including those with whiplash-associated disorders (WAD), the guidelines suggest, with weak strength of evidence, the following interventions: patient education and advice (focusing on assurance, encouragement, prognosis, and pain management), mobilization combined with an individualized, progressive submaximal exercise program, and transcutaneous electrical nerve stimulation [28].

Moreover, a recent systematic review [29] identified seventeen contemporary clinical guidelines for neck and low back pain from eight European countries. For neck pain, high-quality guidelines consistently recommended the following evidence-based treatments: reassurance, advice, and education, manual therapy combined with other interventions, referral for exercise therapy/programs, a variety of oral analgesics and topical medications, and psychological therapies or multidisciplinary care for specific patient subgroups. Other complementary treatments included painkillers, such as paracetamol, NSAIDs (for acute pain only), opioids (for acute pain only), and medications for neuropathic pain. However, all these recommendations were based on weak evidence, primarily derived from expert opinion in high-quality guidelines and/or multiple low-quality guidelines.

On the other hand, recent systematic reviews, in line with the most current clinical guidelines, have demonstrated the positive clinical effects of both active and passive physical therapy treatments for neck pain. For instance, a Cochrane review [30] showed that exercise therapy has beneficial effects for cervical pain in the medium and long term, even after the initial treatment. Moreover, moderate evidence supports the effectiveness of cervico-scapulothoracic and upper extremity strengthening exercises for immediate pain reduction following treatment [30].

Another example is the current systematic review by Castelini [31], which included a total of 119 randomized controlled trials (RCTs) (12,496 patients; 32 interventions) to investigate the most effective conservative treatments for patients with non-specific chronic neck pain. The review found that a combination of active and passive treatments (e.g., exercise and manual therapy) or two passive modalities (e.g., dry needling and manual therapy) may be among the most effective options for reducing pain and disability for up to 3 to 6 months of follow-up, compared to inert treatments. However, the evidence was highly uncertain, leading the authors to recommend further high-quality and larger trials to improve the certainty of the evidence.

Finally, it has been shown that integrating pain neuroscience education leads to a reduction in kinesiophobia and pain anxiety, while also positively affecting pain beliefs, coping strategies, perceptions of physical harm, and medication use in the short term for patients with mechanical neck pain [32]. It is recommended that trigger point dry needling could be incorporated within a pain neuroscience framework, alongside pain neuroscience education, manual therapy, and graded exercises [22].

In clinical guidelines and systematic reviews evaluating various treatment modalities for neck pain, dry needling is recognized as a valid therapeutic option, both as a standalone intervention and in combination with other therapies.

### 1.11. Systematic Reviews of Dry Needling Effectiveness in Neck Pain

Several systematic reviews with meta-analyses have been published in the last 10 years, demonstrating varying levels of effectiveness for DN in the treatment of NP.

In 2023, Hernández-Secorun et al. [25] found high-to-moderate evidence supporting DN as an adjunctive treatment in physical therapy for improving pain levels and disability in the short term, compared to other techniques, such as ultrasound, manual therapy, and stretching alone, as well as DN combined with physical therapy.

In 2022, Navarro-Santana et al. [33] investigated the effects of DN versus MTrP injections (wet needling) applied to MTrPs in the neck–shoulder muscles of subjects with NP. Their research found limited evidence to support the use of lidocaine injections for the treatment of musculoskeletal NP.

In 2021, De las Peñas et al. [34] examined the effects of combining DN with other physical therapy treatments versus the application of these treatments or DN alone on MTrPs in subjects with NP. All studies included were rated with high methodological quality (≥6 points). The authors concluded that there is low-to-moderate evidence supporting the benefits of adding DN to physical therapy treatments for improving pain intensity in the short and medium term, as well as for reducing disability in the short term.

The systematic review by Lew et al. [35] in 2021 compared the effectiveness of DN and manual therapy in MTrPs. The results indicated that both DN and trigger point manual therapy similarly improved pain and function in the short and medium term, with neither treatment showing superiority over the other. Evidence regarding the long-term effects of both therapies was insufficient.

All these systematic reviews provide varying levels of scientific evidence supporting the effectiveness of DN as an adjunctive technique and as a standalone treatment for reducing pain and disability in patients with neck pain.

### 1.12. Electromyographic Assessment for Muscular Activity

**Figure 1:**
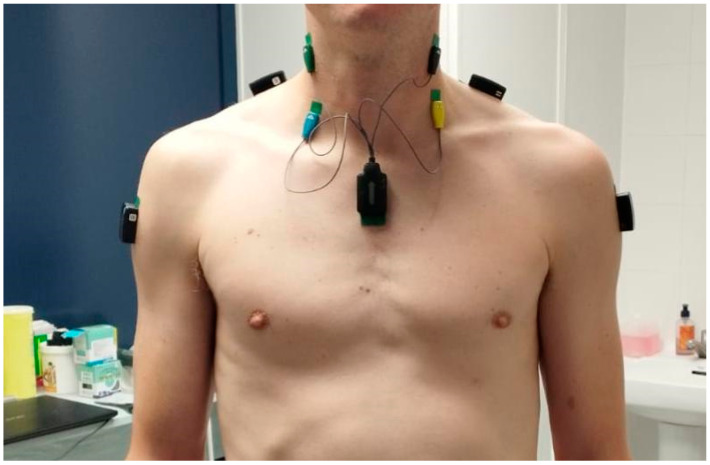
Example of surface electrode placement in deltoid, upper trapezius, anterior scalenus, and sternocleidomastoid, bilaterally.

The neuromuscular activity associated with muscle contraction generates electrical currents that can be recorded and represented by the electromyography signal. Surface EMG captures the signals from various motor units using detectors placed on the skin’s surface (Figure 1). The primary interests in utilizing EMG signals in research include clinical diagnosis and biomedical applications [36].

Electromyography is a method that provides data on muscle activation patterns, including the degree of activation, force level during contraction, and timing of muscle activation. All these parameters are essential for obtaining a comprehensive scientific understanding of motor tasks [37].

For instance, the activity of paraspinal muscles during various tasks, including static postures, dynamic movements, and specific exercises, can be assessed using the versatility of EMG. This enables a deeper understanding of their function in daily activities as well as in pathological conditions [38].

Two common processing techniques, time domain analysis and frequency domain analysis, provide valuable and meaningful data regarding muscle function and performance [38].

Time domain analysis involves examining the temporal characteristics of EMG signals to understand muscle function. Two commonly used parameters are integrated electromyography and root mean square (RMS). Integrated EMG is valuable for quantifying overall muscle activation levels and is defined as the area under the curve of the rectified EMG signal [39]. The RMS calculation is considered to provide significant insight into the amplitude of the EMG signal [40] and reflects the level of physiological activity within the motor unit during contraction [41]. Additional parameters include relative rest time (RRT), which indicates the proportion of time spent below a threshold [42], and muscle activation onset time, a physiological variable related to the onset of contraction in a specific muscle [43]. Muscle onset latency refers to the interval of time between an electrical stimulus and the onset of the muscle’s mechanical response during motor activity [44]. In contrast, peak latency represents the interval of time between an electrical stimulus and the peak amplitude of the muscle’s mechanical response during motor activity [45].

Frequency domain analysis involves examining the spectral characteristics of electromyographic signals. The mean power frequency represents the average frequency weighted by the power spectrum of the EMG signal. This metric provides insights into the frequency characteristics of muscle activity and is associated with muscle fiber type distribution and fatigue. The median frequency denotes the frequency below which 50% of the total power spectrum is contained. During muscle fatigue, median frequency values typically decline due to changes in the recruitment and firing rates of motor units. By monitoring the median frequency, researchers and clinicians can gain valuable information about the onset and progression of muscle fatigue, as well as the adaptability of neuromuscular control strategies [38].

Additionally, other EMG techniques, such as computational methods including normalized mutual information (NMI) and synergy analyses, have been employed to investigate muscle coordination and the effects of lower back or neck–shoulder pain on muscle function [46,47]. The NMI serves as an index that reflects functional connectivity between paired muscles by quantifying their coordination patterns [46].

The term maximal voluntary contraction (MVC) refers to the maximal force produced by a subject when provided with force feedback and verbal encouragement, and when the subject perceives that he has exerted maximal effort. Another technique to assess maximal force from a muscle group is the twitch interpolation technique, which utilizes both voluntary and reflex drives during an attempted MVC. This method involves delivering a supramaximal electrical stimulus to the nerve trunk or intramuscular nerve fibers innerving the target muscle during voluntary contraction. The responses evoked by this stimulus are referred to as “interpolated” twitches, which are superimposed on the level of voluntary torque at the time of stimulation. Known as “twitch interpolation”, this technique confirms that all relevant motoneurons have been recruited and are contributing to the production of maximal force [48].

The degree of muscle activation, associated contraction force, and timing of activation are crucial for executing movement tasks. From a scientific perspective, understanding these factors enhances our comprehension of motor tasks. It is anticipated that non-invasive EMG methods will become increasingly significant in movement science, driven by ongoing technical advancements and a deeper understanding of the relationship between EMG and movement execution [37].

By evaluating muscle activation patterns and coordination in patients with chronic NP, clinicians can gain valuable insights into the neuromuscular dysfunctions associated with this condition. This information can inform targeted interventions and rehabilitation strategies, ultimately improving functional outcomes for these patients. Therefore, we propose this narrative review to provide an update on the current evidence regarding pathological electromyographic activity in individuals with NP and to determine the effectiveness of DN in improving these muscular dysfunctions.

## 2. Materials and Methods

The research was conducted using the following databases: PubMed, Alcorze (search tool of the University of Zaragoza that simultaneously searches the main databases), and Google Scholar. Included studies comprised randomized clinical trials, systematic reviews, narrative reviews, cross-sectional, case–control, cohort, and quasi-experimental studies that investigated electromyographic findings in patients with NP or examined the effects of dry needling on muscular outcomes measured by electromyography in the neck or other body regions. Letters to the editor, single-case series, and single-case reports were excluded from the review. No restrictions were applied regarding date or language.

Firstly, the search terms used in PubMed for pathological electromyographic findings were (electromyography OR altered electromyography OR muscle activity) AND (neck pain OR chronic neck pain OR non-specific neck pain OR mechanical neck pain), yielding a total of 1839 results (up to 13 August 2024). The abstract was reviewed if the title identified cervical pain as the study population and referenced a muscle, muscular electrical activity, or alterations in EMG. All selected abstracts were assessed to determine whether neck pain was a required condition for subject inclusion and whether altered motor patterns or muscle force were studied as primary or secondary outcomes. Additionally, it was evaluated whether changes in muscle force, coordination, fatigue, etc., were measured using EMG recordings. No control group was required for inclusion; however, articles were excluded if they only included healthy subjects. After reviewing the titles and abstracts, 58 articles were included.

Secondly, the search terms used in PubMed related to the effectiveness of dry needling for normalizing muscular activity were: (dry needling) AND (electromyography OR electromyographic muscle activity OR muscle electromyography), resulting in 67 articles. The abstract was reviewed if the title mentioned cervical pain as the study population and referred to a muscle, muscular electrical activity, alterations in EMG, and dry needling. All reviewed abstracts had to include dry needling as the primary treatment and had to measure muscle EMG activity as a primary or secondary outcome. However, due to the limited number of studies investigating EMG as an outcome measure for dry needling in neck pain, interventions involving dry needling in other body regions were also included to strengthen the evidence regarding its effectiveness in enhancing muscle EMG parameters. Based on these criteria, a total of 7 articles were ultimately included.

## 3. Pathological Electromyographic Activity Findings in Neck Pain Patients

We identified multiple studies in the scientific literature that investigated pathological electromyographic activity in the cervical, thoracic, and shoulder muscles of individuals with NP or chronic neck pain (CNP) compared to HS. In the following summary, we found it useful to divide the results into different subsections based on the experimental procedures used to obtain EMG measurements. The EMG assessments were conducted across various tests, including functional tasks (computer and smartphone typing, general upper limb tasks, posture tasks, etc.), and tasks involving cervical force, cervical movement, and the cranio-cervical flexion test (CCFT). The results are reported, classified for each typology of task.

### 3.1. Studies Using Functional Tasks

Among the studies on functional tasks, we subdivided the tasks into seven categories: computer typing task, posture or postural control tasks, general upper limb tasks, violin-playing tasks, jaw clenching tasks, repetitive tasks, and tasks associated with a working day.

#### 3.1.1. Computer Typing Task

Regarding computer and smartphone typing tasks, seven articles were included (Table 1): Threesittidath et al. [49], Kelson et al. [50], Xie et al. [51], Wegner et al. [52], Strom et al. [53] Johnston et al. [54], and Voerman et al. [55]. Overall, these studies reveal altered patterns of upper trapezius (UT) activity during computer typing tasks and at rest in individuals suffering from NP.

#### 3.1.2. Posture or Postural Control Task

The following two studies focus on the influence of postural control and different postures on myoelectrical activity: Cheragh et al. [56] and Namwongsa et al. [57] (Table 1). According to the results of these studies, in patients with CNP, cervical extensors (CE) and UT activity increased following computer typing in both slumped and forward head postures.

#### 3.1.3. General Upper Limb Task

In the literature, we identified 17 studies (Table 2) that conducted research utilizing general upper limb movement tasks as part of their experimental procedures: Wannaprom et al. [58], Wolff et al. [59], Hsu et al. [60], Ghaderi et al. [61], Tsang et al. [62], Januario et al. [63], Christensen et al. [64], Bech et al. [65], Castelein et al. [66], Zakharova-Luneva et al. [67], Falla et al. [68], Andersen et al. [69], Falla et al. [70], Falla et al. [71], and Nederhand et al. [72], In 2002, Nederhand et al. [73] and Larsson et al. [74]’s studies identified multiple alterations, such as higher levels of activity, prolonged muscle activation, delays in muscle activation, and accelerated fatigue in neck muscles during and after general upper limb tasks.

#### 3.1.4. Gait Task

We identified one study (Table 3) that measured neck muscle activity during a gait task: Jimenez Grande et al. [75]. The results indicated that fundamental muscle networks were altered, allowing for the classification of individuals based on differences in neck muscle behavior, particularly in sternocleidomastoid (SCM) and scalene.

#### 3.1.5. Violin Task

One study (Table 3) utilized a violin task as part of its experimental procedure: Kok et al. [76]. The outcomes indicated that violinists with NP exhibited increased muscle activity in the SCM, UT, and left deltoid muscles while playing, as well as higher incidence of simultaneous contraction of both SCM muscles.

#### 3.1.6. Jaw Clenching Task

Jaw clenching tasks were utilized in two studies by Testa et al. to measure the activity of masticatory muscles: Testa et al. [77] and Testa et al. [78] (Table 3). The studies demonstrated that individuals with NP exhibited a different distribution of masseter muscle activity, as well as higher levels of masseter muscle activity compared to HS.

#### 3.1.7. Repetitive Task

Two studies utilized repetitive tasks to evaluate neck muscle activity: Falla et al. [79] and Veiersted et al. [80] (Table 3). These results highlighted that pain altered the amplitude of UT activity, shifted the center of the activity position, and reduced the frequency of EMG gaps during repetitive work tasks.

#### 3.1.8. Working Day Task

Hanvold et al. [81] and Carlson et al. [82] selected the assessment of muscle activity during a working day for their experiments (Table 3). These studies highlighted contradictory findings, with some findings indicating that sustained muscle activity may be a significant predictor of pain and other findings, suggesting no direct correlation between muscle activity and pain levels.

### 3.2. Cervical Force

We found five studies (Table 4), which focused on cervical force measurements to identify potential muscular pathological findings in subjects with CNP: Girasol et al. [83], Schomacher et al. [84], Falla et al. [85], Lindstrom et al. [86], and Falla et al. [87].

During the evaluation of cervical force, it was observed that higher levels of coactivation of the SCM and scalene were associated with reduced neck strength, as well as with increased pain intensity and disability. Finally, a reduction in the neuromuscular efficiency of the SCM and anterior scalene (AS) was also detected.

### 3.3. Cervical Movement

Nine additional studies utilized cervical movement as a tool to assess muscular impairments: Nobe et al. [88], Shamsi’s et al. [89], Lascurain et al. [90], Tsang et al. [62], Park et al. [91], Pinheiro et al. [92], Zabihhosseinian et al. [93], Vikne et al. [94], and finally, in 2010, Cheng et al. [95] (Table 4).

In summary, the results indicate that patients with NP exhibited higher activity in the UT and SCM muscles, along with prolonged activity during flexion–extension and rotation movements compared to HS. The flexion relaxation phenomenon (FRP) in cervical erector spinae was diminished and initiated later during cervical flexion. Additionally, the ratio of flexor to extensor activity was larger in CNP, and cervical extensors activation was delayed on the painful side.

### 3.4. Cranio-Cervical Flexion Test

Finally, we identified nine authors in the scientific literature who assessed electromyographic muscular impairments in individuals with neck pain through the widely used cranio-cervical flexion test (CCFT): Bragatto et al. [96], Iliopoulos et al. [97], Bonilla et al. [98], Jull et al. [99], Steinmetz et al. [100], O’Leary et al. [101], Falla et al. [68], Johnston et al. [102], and Falla et al. [103] (Table 5).

The most notable results were that activity of superficial flexors (SCM and anterior scalene), splenius, and UT was increased and associated with reduced activity of the deep cervical flexors (DCF) in individuals with NP compared to HS.

### 3.5. A Systematic Review of Scapular Muscle EMG Activity in Patients with Idiopathic NP

We identified only one systematic review that assessed cervico-scapular muscle activity in patients with NP: the systematic review by Castelein et al. [104] (Table 5).

## 4. Outcomes of Neck Muscle Activity, Measured by Electromyography, in Individuals with Neck Pain or Healthy Subjects Following Dry Needling

We now turn to the analysis of the current evidence regarding DN outcomes on muscular activity. We identified three studies in the literature that investigated the effects of DN on the myoelectrical activity of neck muscles in individuals with NP and HS (Table 6).

In 2022, Rodríguez-Jiménez et al. [105] compared the effects of DN and manual pressure release on an active MTrP in the UT on CCFT performance, pressure pain threshold, and cervical range of motion in patients with CNP. The interventions consisted of a single session of DN targeting the UT active MTrPs in a supine position or a single session of manual trigger point pressure release over the UT for 30 s. Regarding neck muscle activity, a decrease in SCM activity during all stages of the CCFT was observed in both groups after treatment. However, no differences were observed between groups at any stage of the CCFT. Additionally, no changes were noted in EMG amplitude for the Scalene or UT muscles.

Moreover, in 2022, Sánchez-Infante et al. [106] measured the effects of DN on latent trigger points in the UT regarding the pressure pain threshold, RMS, and mean frequency of electromyography in 23 HS with latent trigger points in the UT. The results were compared to those of a sham DN in 23 HS. UT EMG activity was evaluated in two different tests: at rest and during isometric contraction of the UT. The results indicated that a single session of DN was effective in reducing the electromyographic activity of the UT 30 min after the intervention, lasting up to 24 h at rest and up to 72 h during isometric contraction. Additionally, muscle fatigue was diminished 72 h post-intervention during isometric contraction.

In 2017, De Meulemeester et al. [107] evaluated the effect of DN of the UT on EMG compared to a no-intervention control group in women with trapezius myalgia, following a typing task. They wanted to determine if the effects were dependent on the occurrence of local twitch responses during DN. Ten minutes after the single session of DN, the results showed a lower increase in UT EMG activity compared to the control group, regardless of the number of local twitch responses induced.

## 5. Outcomes of Dry Needling Studies on Muscular Activity Measured by Electromyography in Other Muscles of Various Populations

We also found four studies in the scientific literature that investigated how the application of DN affects electromyographic activity levels in other muscles across various populations (Table 6).

For example, Schneider et al. [108] investigated whether dry needling of MTrPs in the gluteus medius in HS improved the strength and muscle activation levels immediately after the intervention. The results demonstrated an increase in muscle force and a reduction in the level of electromyographic activation immediately after the intervention during the maximal voluntary isometric contraction of the gluteus medius.

Additionally, Benito et al. [109] conducted a study with triathletes to compare the immediate effects of DN and ischemic compression on MTrPs from medial and lateral gastrocnemius during treadmill walking at different speeds. The results showed a reduction in EMG activity in the DN group compared to the ischemic compression technique at a speed of 1 m/s immediately after both treatments, but no differences were observed at speeds of 1.5 m/s or 2.5 m/s (*p* = 0.037).

Another study of López González et al. [110] compared the effects of DN against placebo DN on the peroneus longus and tibialis anterior in basketball players with chronic ankle instability. The DN group showed an increase in the pre-activation values for the peroneus longus and tibialis anterior during a landing task, with this increase maintained one month after treatment. Furthermore, improvements in static postural control measures were observed in the DN group.

Finally, Wang’s study [111] measured the effects of DN on the myoelectric activity of the lumbosacral multifidus in adults with low back pain. The results indicated no difference in electromyographic amplitude of the lumbar multifidus immediately after DN or one week after DN.

## 6. Discussion

A multimodal approach is recommended, with moderate strength of evidence, for patients with chronic neck pain and mobility deficits. This approach should include dry needling, along with other therapies [28]. Furthermore, the combination of active and passive treatments, or two passive modalities, such as dry needling and manual therapy, may be among the most effective options for reducing pain and disability for up to 3 to 6 months of follow-up, compared to inert treatments [31]. However, the evidence remains highly uncertain. Furthermore, a recent randomized controlled trial demonstrated that pain neuroscience education, combined with trigger point therapy and manual therapy, is more effective in reducing kinesiophobia and pain and positively impacts pain beliefs, pain coping strategies, physical harm perception, and medication use in the short term for patients with mechanical neck pain [32].

On the one hand, these RCTs, systematic reviews, and clinical guidelines [28,29,31,32] demonstrate that dry needling primarily improves pain and disability. However, other clinically relevant variables, such as muscle strength and function, remain understudied; whereas, the degree of muscle activation, associated contraction force, and timing of activation are critical for performing movement tasks [34]. Moreover, among these studies, when muscle force is studied, no electromyography measurements are reported, despite the fact that EMG is a valid and useful technique that provides valuable patterns of muscle force production and function.

On the other hand, chronic neck pain or mechanical neck pain lacks an identifiable pathological basis as the underlying cause of the symptoms [2]. Therefore, it is crucial to understand the factors that may contribute to mechanical neck pain, including muscle dysfunctions.

Due to the need to explore mechanisms and the effectiveness of dry needling, as well as its impact on muscle force production measured by EMG in patients with neck pain and considering the importance of enhancing our understanding of the altered muscle patterns involved in neck pain, this narrative review was conducted to contribute new insights into these areas.

After reviewing the scientific literature, we identified fifty-eight studies that investigated altered muscle control patterns in patients with neck pain, including chronic neck pain. However, only seven studies investigated the efficacy of dry needling interventions using EMG to measure muscle performance under various conditions, of which three specifically focused on dry needling for neck pain.

Concerning pathological electromyographic findings, the studies demonstrated several alterations in cervical muscle motor patterns during various tasks, including longer continuous activation durations of neck and shoulder muscles, increased muscle activity, greater and earlier onset of neck muscle fatigue, alterations in activation patterns, delayed peak muscle activation, changes in muscle network coordination, the altered distribution of muscle activity, and increased resting muscle activity. The evidence found was primarily composed of case control, cross-sectional, and cohort studies. Several limitations can be attributed to these study designs. For instance, cross-sectional studies are useful for identifying associations but make it difficult to establish causality. This limitation prevents us from directly attributing motor dysfunctions as the cause of neck pain. Additionally, other limitations of the reviewed studies include heterogeneity in methodologies, such as variations in pain and disability levels reported among neck pain subjects, differences in the duration of pain experienced, small sample sizes, lack of standardization in postures during tasks, inconsistency in task performance, the absence of assessments for psychosocial factors, like kinesiophobia or anxiety, and an overemphasis on the trapezius muscle, while potentially relevant muscles, such as the scalene or sternocleidomastoid (SCM), may not have been adequately assessed.

Concerning dry needling studies, two randomized controlled trials and one laboratory study assessed EMG parameter measurements in patients with neck pain after a single session of dry needling, but they reported contradictory and inconsistent findings. Other randomized controlled trials conducted in different body regions have shown that a single session of dry needling enhances muscle force production [108], reduces muscle EMG activation levels [109] during contraction, and improves postural control [110]. However, one study did not find improvements in muscle function [111].

We have identified several limitations in the studies mentioned: only latent myofascial trigger points (MTrPs) were treated, while active MTrPs, which are more commonly associated with the pain experienced by the patient, were not targeted. Given that pain levels have been associated with altered neck muscle activity [62,68,81,86,101], we hypothesize that targeting active MTrPs may be more relevant than targeting latent MTrPs. Additionally, in the studies reviewed, only one session of dry needling was applied; it is possible that the effects on muscle performance would be more pronounced with multiple sessions, as demonstrated in other high-quality randomized controlled trials [25,34,35,112]. Furthermore, only one latent MTrP in the trapezius was treated, while studies on the prevalence of MTrPs indicate that other muscles also present active trigger points in neck pain patients [9]. After reviewing the literature, no studies were found that reported the efficacy of dry needling on muscular electromyography outcomes at mid- and long-term follow-up. Finally, the narrative review approach, itself, has some limitations, such as the absence of a statistical synthesis of the results.

We deem it relevant for future research to determine the effects of dry needling as a standalone intervention or in comparison to other techniques on muscular EMG outcomes in the medium and long term in patients with neck pain. Additionally, the effects of multiple dry needling sessions on muscle performance should be explored. Future studies should prioritize high-quality research with larger sample sizes and reduced bias. To date, the majority of studies have focused on the upper trapezius, but other neck and shoulder muscles, including the scalene, SCM, and cervical extensors, should also be investigated.

## 7. Conclusions

On the one hand, the current scientific evidence supports variable electromyographic pathological findings in individuals with CNP. Based on the studies reviewed and the types of tasks performed, we can conclude the following:

During computer typing tasks, the conflicting results reported prevent definitive conclusions regarding alterations in muscle activity. However, it is plausible that individuals with CNP may exhibit increased levels of activity in the CE, AS, and UT, along with a diminished ability to relax the UT after these tasks compared to HS.

Regarding smartphone use, altered muscle activity varies depending on body position. In CNP patients, CE and UT activity is higher in slumped and forward head postures. Additionally, there is an association between neck flexion angle, range of motion, and muscle activity.

During general upper limb tasks, individuals with neck pain exhibit higher activity levels in the CE, UT, SCM, TE, SA, and pectoralis minor muscles during or after the task. Prolonged muscle activation of the UT, CE, SCM, and TE is also observed. Additionally, there is a delay in DCF activity and accelerated fatigue of the UT in CNP patients.

In tasks involving violin playing, violinists show increased activity in the SCM, UT, and deltoid muscles.

During jaw clenching tasks, individuals with NP demonstrate a different distribution of masseter muscle activity, along with higher overall levels of masseter activity.

In repetitive work tasks, studies highlight that pain alters the amplitude, shifts the center of activity, and reduces the frequency of EMG gaps in the UT muscle.

During daily work activities, contradictory findings regarding UT activity levels are observed in individuals with NP.

During cervical force tasks, reduced and less defined activity in the SCM is noted, along with increased coactivation of the SCM and SC, which is associated with reduced neck strength and greater pain intensity and disability. Furthermore, a decrease in neuromuscular efficiency of the SCM and AS is detected, along with a reduction in the modulation of the discharge rate of individual motor units in the SCM.

During cervical movement tasks, such as flexion–extension and rotation, higher activity levels in the UT and SCM are observed, along with prolonged activity in the UT. The FRP in the CE is diminished and begins later during cervical flexion. Additionally, during flexion–extension movements, the ratio of flexors to extensors is larger in CNP patients. The onset activation of the CE is delayed on the painful side, and generally, altered EMG patterns are demonstrated during cervical movements.

Finally, in the CCFT, increased electromyography activity in the superficial flexors (SCM, AS, and splenius capitis) and UT is associated with reduced EMG activity of the DCF in individuals with neck pain compared to asymptomatic subjects. Furthermore, higher levels of NP are correlated with decreased activity in the DCF and increased activity in the AS, SCM, and UT muscles.

On the other hand, after reviewing the literature on the outcomes of dry needling studies regarding muscular activity measured by electromyography in various populations, we reached the following conclusions:

DN applied to an active TrP in the UT can be suggested as an intervention to enhance the performance of the CCFT.

DN applied to latent MTrPs in the UT after typing tasks in HS resulted in decreased UT activity and fatigue in the short term.

In women with trapezius myalgia, DN applied to the UT led to a lower increase in EMG activity compared to no intervention.

## Figures and Tables

**Table 1:** Findings of pathological electromyographic activity in neck pain patients during the evaluation of typing and posture tasks.

First Author and Year	Type of Study Sample Size	Limitations	Conclusions
**Typing tasks studies**			
Threesittidath [49] 2024	CS *n* = 40	Subjects reported an average pain level classified as moderate.	In subjects with NP, there are neuromuscular deficits in SP and AS.
Kelson [50] 2017	CC *n* = 13	Small sample size.	Workers with NP tend to have longer continuous durations of trapezius muscle activation.
Xie [51] 2016	CC *n* = 40	Only one position was used to measure the typing task.	Symptomatic subjects exhibited increased muscle activity in the neck and shoulder region while texting on a smartphone.
Wegner [52] 2010	CC *n* = 38	Scapular position was only visually assessed.	Exercises aimed at scapular correction may effectively modify the aberrant trapezius activation pattern in individuals with NP.
Strom [53] 2009	CC *n* = 52	Medication intake by participants was not recorded.	In CNP patients, pain is associated with trapezius vasodilation but not with EMG activity.
Johnston [54] 2008	CS *n* = 85	Correlation between level of pain and EMG was not assessed.	Computer workers, both with and without NP, show increased EMG activity in cervical flexors and extensors compared to non-working females.
Voerman [55] 2007	CC *n* = 41	WAD had significantly higher weight compared to WMSD.	There is no definitive for abnormal muscle activation patterns in WMSD and WAD.
**Posture task studies**			
Cheragh [56] 2024	CS *n* = 15	Only the slump position was used for computer typing.	Sixty minutes of computer typing in the slump posture increased the activity of the neck musculature.
Namwongsa [57] 2019	CC *n* = 44	Short duration of the typing task.	NP smartphone users demonstrated slightly higher activity in their neck muscles than asymptomatic users.

AS: anterior scalene; CNP: chronic neck pain; CC: case control; CS: cross-sectional; EMG: electromyography; NP: neck pain; SP: serratus posterior; WAD: whiplash-associated disorders; WMSD: work-related musculoskeletal disorders.

**Table 2:** Findings of pathological electromyographic activity in neck pain patients during evaluation of general upper limb task.

First Author and Year	Type of Study Sample Size	Limitations	Conclusions
**General upper limb task studies**			
Wannaprom [58] 2023	CC *n* = 90	The control of scapular position during the task was not assessed.	NP subjects had higher neck extensors activity in all tasks and higher UT/LT and UT/SA ratios in a few tasks at low force level.
Wolff [59] 2022	CS *n* = 36	The EMG data were not obtained during dynamic movements	CNP subjects exhibited lower upper trapezius activation during bilateral reaches.
Hsu [60] 2020	CC *n* = 50	Young sample between 20–30 years old.	Neck muscle fatigue may significantly influence the timing of muscle activation and trigger a protective control strategy in neck muscles during shoulder flexion.
Ghaderi [61] 2019	CS *n* = 40	A score less than 4 in VAS for neck pain was established as an inclusion criteria.	There were relationships between CNP and onset delays in the anterior and middle deltoid and a peak delay in the UT.
Tsang [62] 2018	CC *n* = 68	There were differences in the time taken to complete the task between the groups.	Significant alterations in the activation patterns of multiple cervical and thoracic muscles were observed in NP compared to asymptomatic.
Januario [63] 2017	CC *n* = 30	The duration of pain was not recorded.	Neck–shoulder pain had no effect on the sEMG patterns, indicating no impaired sEMG activity in individuals with neck–shoulder pain.
Christensen [64] 2017	CC *n* = 50	Scapular control and orientation were not assessed.	Scapular movement elicited different muscle activity responses between individuals with NP and the control group.
Bech [65] 2017	CC *n* = 20	No information regarding the subjects’ usual activity levels was recorded.	Following stimulation of the accessory nerve, subjects with NP reported a 20% lower MVC in all three portions of the trapezius compared to the control group.
Castelein [66] 2016	CC *n* = 38	The influence of pain has not been sufficiently considered.	Patients with NP and scapular dyskinesis showed lower MT activity compared to healthy controls.
Zakharova-Luneva [67] 2012	CC *n* = 38	The upper limb task had a short duration of 10 s.	Changes in the activity patterns of the trapezius muscle are evidenced in patients with CNP and scapular dysfunction.
Falla [68] 2011	CS *n* = 32	Other disability questionnaires could have been considered.	The delay and altered activity in DCF in CNP patients are associated with intensity of neck pain.
Andersen [69] 2008	CS *n* = 62	Due to limited statistical power, differences in muscle torque below 10% could not be detected.	CNP is associated with decreased strength capacity and lower trapezius activity, especially during slow concentric and eccentric contraction.
Falla [70] 2004	CC *n* = 20	Small sample size.	Aberrant muscle activity may result from an altered motor strategy aimed at reducing the activity of painful muscles.
Falla [71] 2004	CC *n* = 22	Other muscles could have altered the EMG signal through the mucosal wall.	Patients with CNP exhibit a delay in the activity of DCF and contralateral SCM and AS during arm flexion compared to controls.
Nederhand [72] 2003	Prospective Cohort *n* = 92	Only the EMG of UT was measured.	In patients with acute whiplash-associated disorders (WAD), changes in upper trapezius (UT) activity were observed, potentially resulting from segmental and supraspinal inhibitory effects.
Nederhand [73] 2002	CC *n* = 55	Only one specific task was performed.	In subjects with WAD grade 2, greater and prolonged UT activation on the active side was observed in response to physical exercise compared to the control group.
Larsson [74] 1999	CC *n* = 96	Only women were included in the control group.	In cases of chronic trapezius myalgia, RMS values were elevated during rest periods as well as during the most intense contractions of arm tasks.

AS: anterior scalene; CC: case control; CNP: chronic neck pain; CS: cross-sectional; EMG: electromyography; DCF: deep cervical flexors; LT: lower trapezius; MT: middle trapezius; MVC: maximal voluntary contractions; NP: neck pain; RMS: root mean square; SCM: sternocleidomastoid; UT: upper trapezius; WAD: whiplash-associated disorders.

**Table 3:** Findings of pathological electromyographic activity in neck pain patients during evaluation of gait, violin, jaw clenching, repetitive, and working day tasks.

First Author and Year	Type of Study Sample Size	Limitations	Conclusions
**Gait task studies**			
Jimenez-Grande [75] 2021	CC *n* = 40	Muscle networks are typically analyzed using a greater number of muscles.	In CNP, fundamental muscle networks were altered, particularly in SCM and scalene.
**Violin task studies**			
Kok [76] 2018	CC *n* = 20	Posture of violinist and positioning of violin were not evaluated.	Increased activity of SCM, UT, and left deltoid was found while playing violin in violinist with NP.
**Jaw clenching task studies**			
Testa [77] 2017	CC *n* = 20	The head position was not standardized between subjects.	People with CNP display higher activity and altered distribution of masseter muscle during jaw clenching.
Testa [78] 2015	CC *n* = 20	Small sample size.	Individuals with NP showed a different distribution of activity and higher levels of activity in the masseter muscle.
**Repetitive task studies**			
Falla [79] 2017	CS *n* = 10	Participants were only 10 young men (age 26.2 ± 3.1).	There is a different distribution of UT activity after performing repetitive lifting task in the presence of pain.
Veiersted [80] 1993	Prospective Cohort *n* = 30	EMG gaps physiological significance is unclear.	EMG gap analysis could serve as a generic risk factor for the development of trapezius pain.
**Working day studies**			
Hanvold [81] 2013	Prospective Cohort *n* = 40	Some of the time-varying variables were not assessed.	Sustained trapezius muscle activity is associated with NP.
Carlson [82] 1966	Prospective Cohort *n* = 20	General activity levels of the subjects during their work were not regulated.	Ambulatory measurements of EMG activity did not differentiate persons with NP from asymptomatic.

CC: case control; CNP: chronic neck pain; CS: cross-sectional; EMG: electromyography; NP: neck pain; SCM: sternocleidomastoid; UT: upper trapezius.

**Table 4:** Findings of pathological electromyographic activity in neck pain patients during evaluation of cervical force and movement.

First Author Year	Type of Study Sample Size	Limitations	Conclusions
**Cervical force**			
Girasol [83] 2017	CS *n* = 40	The sample was composed by young age subjects (24.31 years) and 38 out of 40 were women.	The decrease in skin temperature over MTrPs in the UT was linked to a lower median frequency both at rest and during isometric contraction, along with an increase in RMS values at rest.
Schomacher [84] 2012	CC *n* = 20	Small sample size.	Individuals experiencing NP show reduced and less defined activity in the semispinalis cervicis muscle.
Falla [85] 2010	CS *n* = 18	Intramuscular EMG was not acquired during circular contraction.	Women with CNP showed a decreased modulation of the discharge rate in individual motor units of the SCM muscle.
Lindstrom [86] 2011	CC *n* = 23	Kinesiophobia could have influenced muscles coactivation.	Increased coactivation of the SCM and splenius capitis muscles is linked to reduced neck strength, elevated pain levels, and greater disability.
Falla [87] 2004	CC *n* = 40	Only cervical flexion force was measured.	In subjects with NP, the neuromuscular efficiency of the SCM and AS muscles was reduced at 25% of MVC.
**Cervical movement**			
Nobe [88] 2022	CC *n* = 48	Variations in demographic characteristics among the groups.	Cervical flexor and extensor activity is increased and associated with an imbalance in activity between these muscles in nonspecific NP.
Shamsi’s [89] 2021	CC *n* = 50	Patients scoring over 50 mm on the VAS were excluded.	The incidence of the flexion relaxation phenomenon was reduced and occurred later in individuals with CNP, indicating prolonged activity of the cervical extensors during flexion.
Lascurain [90] 2018	CC *n* = 40	The influence of pain and kinesiophobia were not measured.	Individuals with neck pain exhibited reduced scalene muscle activity during both cervical flexion and extension.
Tsang [62] 2018	CC *n* = 64	Differences in time taken to complete the tasks between the groups.	In subjects with NP, distinct activation patterns of the cervical and thoracic muscles were observed, which were linked to pain and disability.
Park [91] 2017	CC *n* = 40	The sample was composed by young age subjects (23.45 and 23.35 years in each group).	Subjects with unilateral posterior NP exhibited greater asymmetry in muscle activation during prone neck extension compared to the control group.
Pinheiro [92] 2016	CC *n* = 60	The timing of day evaluations was not standardized.	Differences in flexion–relaxation ratios were observed between the groups.
Zabihhosseinian [93] 2015	CC *n* = 25	The neck pain levels of participants were not recorded.	Subjects with mild to moderate NP exhibited differences in neuromuscular control and experienced myoelectric fatigue sooner.
Vikne [94] 2013	CC *n* = 30	Intermediate sample size.	In patients with chronic WAD, normal muscle activation levels were observed for specific velocities and displacements.
Cheng [95] 2010	CC *n* = 24	The ROM was recorded within a pain-free range.	There is a suggestion that altered EMG patterns occur in CNP patients during voluntary sagittal neck movements.

AS: anterior scalene; CC: case control; CNP: chronic neck pain; CS: cross-sectional; EMG: electromyography; MVC: maximal voluntary contractions; MTrPs: myofascial trigger points; NP: neck pain; RMS: root mean square; ROM: range of motion; SCM: sternocleidomastoid; UT: upper trapezius; VAS: visual analog scale; WAD: whiplash-associated disorders.

**Table 5:** Findings of pathological electromyographic activity in neck pain patients during evaluation of cranio-cervical flexion test and multiples tasks.

First Autor and Year	Type of Study Sample Size	Limitations	Conclusions
**Cranio-cervical flexion test**			
Bragatto [96] 2023	CC *n* = 100	Pain levels during the CCFT test were not recorded.	Regardless of the presence of neck pain, poor cervical muscle performance was observed in women with CNP and with migraine.
Iliopoulos [97] 2022	CC *n* = 44	Deficits in ROM of cervical spine were not assessed.	In subjects experiencing moderate pain, disability, and pain duration, no significant changes in EMG activity of the SCM were observed during the CCFT.
Bonilla [98] 2020	CC *n* = 60	Only women were included in the sample.	Women with CNP showed higher activity of superficial neck flexors and extensors during the CCFT compared to asymptomatic women.
Jull [99] 2016	CS *n* = 32	Only women were included in the sample.	Increased activity in the superficial flexor muscles indicated a decrease in DCF activity during the CCFT.
Steinmetz [100] 2016	CC *n* = 54	High inter-subject variability was found in muscle activity recordings.	Violinists experiencing neck pain related to playing exhibited increased activity of the SCM muscle during the CCFT.
O’Leary [101] 2011	Cohort Retrospective *n* = 84	Only the average pain intensity experienced over the past week was assessed.	A positive relationship was found between pain intensity and superficial muscle activity during CCFT.
Falla [68] 2011	CS *n* = 32	Other disability questionnaires could have been considered.	Higher levels of pain were associated with lower activity of DCF during CCFT.
Johnston [102] 2008	CC *n* = 85	Socioeconomic status or comorbidity were not assessed.	Female office workers with NP showed increased activity in the superficial neck flexors during CCFT.
Falla [103] 2004	CC *n* = 20	Cross talk from other cervical flexor muscles could have biased the results.	Lower EMG amplitudes in DCF were associated with higher values in the superficial neck muscles during CCFT.
**Multiple tasks analyzed in a systematic review**			
Castelein [104] 2015	SystematicReview	Only studies that analyzed EMG amplitude, timing, and fatigue were included.	There are no significant differences in the mean EMG amplitude of the UT during rest and activities performed below shoulder level. Additionally, no conclusions can be made regarding scapular EMG amplitude during overhead activities.

CC: case control; CS: cross-sectional; CCFT: cranio-cervical flexion test; CNP: chronic neck pain; DCF: deep cervical flexors; EMG: electromyography; NP: neck pain; ROM: range of motion; SCM: sternocleidomastoid; UT: upper trapezius.

**Table 6:** Outcomes of dry needling studies regarding EMG muscle activity.

First Author and Year	Type of Study Sample Size	Limitations	Conclusions
**Dry needling and EMG outcome on neck muscles**			
Rodríguez-Jiménez [105] 2022	RCT *n* = 50	No sham group was part of the study.	A single session of DN or manual pressure applied to the UT active MTrP has a limited impact on muscle performance during the CCFT in individuals with CNP.
Sánchez-Infante [106] 2022	RCT *n* = 46	The population studied consisted of healthy volunteers, and only short-term effects were evaluated.	A single session of DN in latent MTrPs was effective in reducing the EMG activity of the UT in the short term compared to the sham group.
De Meulemeester [107] 2017	Controlled laboratory study *n* = 24	Only short-term effects and trapezius muscle were assessed.	A single DN session led to a lower increase in EMG activity of UT compared to no intervention.
**Dry needling and EMG outcome on other body region**			
Schneider [108] 2022	RCT *n* = 39	Measures were taken only immediately after the application of DN, and no functional movements were evaluated.	A single session of DN targeting latent MTrPs in the gluteus medius enhances force production and reduces the muscle EMG activation level required during contraction.
Benito [109] 2021	RCT *n* = 34	Pain levels after DN treatment were not assessed.	A single session of DN targeting latent MTrPs in the gastrocnemius muscle of triathletes reduces the muscle’s EMG at a speed of 1 m/s.
López González [110] 2021	RCT *n* = 32	There was not a control group of asymptomatic subjects.	In basketball players with chronic ankle instability, a single session of DN applied to latent MTrPs in the tibialis anterior and peroneus longus muscles increases muscle pre-activation during landing tasks and enhances static postural control.
Wang [111] 2020	RCT *n* = 44	The subjects’ activity levels and medication intake were not evaluated.	A single session of DN may not be adequate to enhance the function of the lumbosacral multifidus.

CCFT: cranio-cervical flexion test; CNP: chronic neck pain; DN: dry needling; EMG: electromyography; MTrPs: myofascial trigger points; RCT: randomized controlled trial; UT: upper trapezius.

## Data Availability

The original contributions presented in the study are included in the article, further inquiries can be directed to the corresponding author.

## Abbreviations

AS	Anterior scalene
CCFT	Cranio-cervical flexion test
CE	Cervical extensors/cervical erector spinae
CNP	Chronic neck pain
CC	Case control
CS	Cross-sectional
DCF	Deep cervical flexors
DN	Dry needling
EMG	Electromyography/superficial electromyography
FRP	Flexion relaxation phenomenon
HS	Healthy subjects
LT	Lower trapezius
MT	Middle trapezius
MTrPs	Myofascial trigger points
MVC	Maximal voluntary contractions
NP	Neck pain
NSNP	Non-specific neck pain
RCT	Randomized controlled trial
RMS	Root mean square
ROM	Range of motion
RRT	Relative rest time
SA	Serratus anterior
SCM	Sternocleidomastoid
SP	Serratus posterior
TE	Thoracic extensors
TrP-DN	Trigger point dry needling
UT	Upper trapezius
WAD	Whiplash-associated disorders
WMSD	Work-related musculoskeletal disorders
VAS	Visual analog scale
